# Maternal Exercise Programs Glucose and Lipid Metabolism and Modulates Hepatic miRNAs in Adult Male Offspring

**DOI:** 10.3389/fnut.2022.853197

**Published:** 2022-03-01

**Authors:** Liyuan Zhou, Shunhua Li, Qian Zhang, Miao Yu, Xinhua Xiao

**Affiliations:** Key Laboratory of Endocrinology, Department of Endocrinology, Translational Medicine Center, Ministry of Health, Peking Union Medical College Hospital, Peking Union Medical College, Chinese Academy of Medical Sciences, Beijing, China

**Keywords:** maternal voluntary wheel running training, glucose and lipid metabolism, transcriptome, miRNAs, offspring, mice

## Abstract

Detrimental exposures in mothers are recognized as risk factors for the development of metabolic dysfunction in offspring. In contrast, maternal exercise has been reported to be an effective strategy to maintain offspring health. However, the mechanisms underlying the protective effects of maternal exercise on adult offspring metabolic homeostasis are largely unclear. This study aims to investigate whether maternal exercise before and during pregnancy could combat the adverse effects of maternal high-fat diet (HFD) on metabolism in 24-week-old male offspring and to explore the role of miRNAs in mediating the effects. Female C57BL/6 mice were fed with either control diet or HFD 3-week prior to breeding and throughout pregnancy and lactation, among whom half of the HFD-fed mice were submitted to voluntary wheel running training 3-week before and during pregnancy. Male offspring were sedentary and fed with a control diet from weaning to 24 weeks. Body weight, the content of inguinal subcutaneous adipose tissue and perirenal visceral adipose tissue, glucose tolerance, and serum insulin and lipids in offspring were analyzed. Hepatic tissues were collected for transcriptome and miRNA sequencing and reverse transcription-quantitative polymerase chain reaction validation. The results showed that maternal HFD resulted in significant glucose intolerance, insulin resistance, and dyslipidemia in adult offspring, which were negated by maternal exercise. Transcriptome sequencing showed that maternal exercise reversed perinatal HFD-regulated genes in adult offspring, which were enriched in glucose and lipid metabolic-related signaling pathways. At the same time, maternal exercise significantly rescued the changes in the expression levels of 3 hepatic miRNAs in adult offspring, and their target genes were involved in the regulation of cholesterol biosynthesis and epigenetic modification, which may play an important role in mediating the intergenerational metabolic regulation of exercise. Overall, our research pioneered the role of miRNAs in mediating the programming effects of maternal exercise on adult offspring metabolism, which might provide novel insight into the prevention and treatment of metabolic disorders in early life.

## Introduction

The worldwide prevalence of type 2 diabetes mellitus (T2DM) and obesity has caused unprecedented challenges on global health and economic burden. As highly heterogeneous diseases, the etiology and pathogenesis of these metabolic disorders are still incompletely understood. Aside from the direct influences of genetics and environmental factors on the susceptibility to developing obesity and T2DM, over the last few decades, substantial evidence has found an association between early-life exposures and adult-onset diseases. Beginning with the “developmental origins of health and disease” (DOHaD) theory put forward by Barker in 1989 (1), also named as “metabolic memory,” extensive epidemiological investigations and experimental animal models demonstrated that intrauterine malnutrition (under-nutrition and over-nutrition) significantly elevated the risk of metabolic disorders in adulthood (2–9). Therefore, maternal lifestyle intervention may be a practical measure to break the intergenerational cycle of chronic metabolic diseases.

Given the specificity and safety of pregnancy, it is quite important to choose an appropriate, feasible, and effective strategy. Exercise has been strongly recommended throughout the treatment of diabetes and prediabetes by multiple guidelines. Although the intensity and duration are still controversial, previous clinical studies have proved that maternal exercise reshaped pregnancy outcomes and future children's health of reproductive-age women with overweight or gestational diabetes (10). Yet, little is known about the long-term consequences of maternal exercise on offspring in human cohorts. In terms of rodent models, our team and other groups have demonstrated that maternal exercise before and during pregnancy alleviated the adverse impact of maternal high-fat diet (HFD) on the metabolic health of adult offspring (11, 12). However, the underlying mechanism responsible for the intergenerational metabolic benefits of maternal exercise has not been fully clarified.

Recently, emerging scientific literatures have confirmed that maternal hyperglycemia and dyslipidemia caused by adverse preconception and gestational lifestyle can affect embryonic or fetal epigenetics and some of these changes are lasting throughout the lifespan, which could be one of the most plausible etiologies of metabolic diseases (13–19). Epigenetic modification regulates gene expression in the absence of changes in the DNA sequence. Recently, microRNAs (miRNAs), a group of small non-coding RNAs with about 21–24 nucleotides, have emerged as a frontier epigenetic mechanism. They sequester target genes for degradation or prevent their translation by specifically binding to the 3′ untranslated regions (3′-UTR) of mRNA and interacting with the Dicer complex. In light of their properties of regulating pancreatic β-cell dysfunction and modulating glucose uptake and transport, several studies have also involved miRNAs in T2DM pathogenesis (20–23). However, the research on the effect of maternal-fetal metabolic interaction on the epigenetic modification of offspring is limited.

In this study, we aim to assess whether maternal exercise 3-week prior to breeding and during pregnancy could counter the harmful metabolic outcomes of perinatal HFD on the next generation. Besides, we would further clarify the role of miRNAs epigenetic modification in mediating the regulation effects of maternal exercise on glucose and lipid metabolism in adult offspring.

## Materials and Methods

### Animal Model

C57BL/6 mice were purchased from the Beijing Vital River Laboratory Animal Technology Co., Ltd. (Beijing, China, SCXK-2016-0006) and were raised in a standard specific pathogen-free environment, having free access to food and water. Females aged 6-week were randomly divided into three groups during 3 weeks before breeding and throughout pregnancy: the control group (C, *n* = 6) with a normal control diet (D12450J) (10% of the calories as fat); high-fat group (HF, *n* = 6) with a high-fat diet (HFD) (D12492) (60% of the calories as fat); high-fat with exercise intervention group (HF-EX, *n* = 6) with the HFD (D12492) and a voluntary wheel running (VWR) training (mice housed in cages containing running wheels and having free access to wheels all day). The running wheels were 13 cm in diameter and 6 cm in width (Yuyan Instruments Co., Ltd, Shanghai, China). They maintained their original diets during lactation.

**Figure 1:**
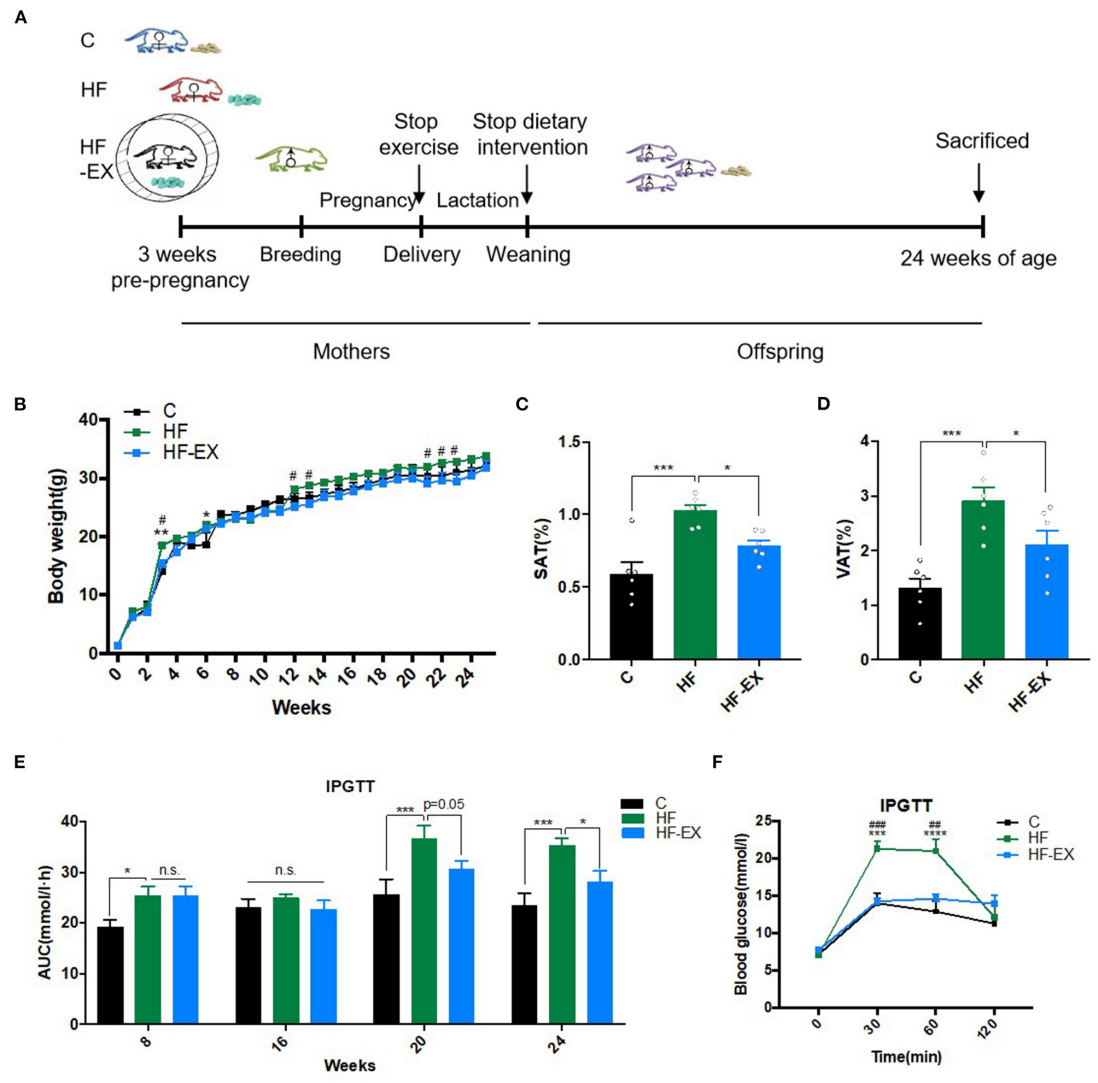
Glucose and lipid metabolism in male offspring. **(A)** Experimental scheme; **(B)** body weight changes; **(C)** subcutaneous adipose tissue mass at 24 weeks; **(D)** visceral adipose tissue content at 24 weeks; **(E)** AUC of blood glucose values during intraperitoneal glucose tolerance tests; **(F)** intraperitoneal glucose tolerance test at 24 weeks. C, offspring of dams fed the normal control diet; HF, offspring of dams fed the high-fat diet; HF-EX, offspring of dams intervened with a high-fat diet and exercise; SAT, subcutaneous adipose tissue mass; VAT, visceral adipose tissue; IPGTT, intraperitoneal glucose tolerance test; AUC, area under the curve. Data are expressed as means ± SD (*n* = 6/group) and are analyzed by one-way ANOVA or two-way ANOVA, with Turkey *post-hoc* analyses. Mean values were significantly different between the groups: *HF vs. C, *p < 0.05, **p < 0.01, ***p < 0.001, ****p < 0.0001; ^#^HF-EX vs. HF, ^#^*p* < 0.05, ^##^*p* < 0.01, ^###^*p* < 0.001.

Females bred with sedentary C57BL/6 males fed with a control diet after the intervention of the first 3 weeks. To ensure no nutritional bias between litters, each litter was culled to five pups for each dam at birth. Male offspring were weaned at postnatal 21-day on a chow diet until 24 weeks. Body weights of male offspring were measured each week. At 24 weeks of age, one male offspring from each litter (*n* = 6 per group) was sacrificed to evaluate the effects of maternal exercise on offspring. We collected the blood samples and livers. The experimental design was shown in Figure 1A. All procedures were approved by the animal care and use committee of the Peking Union Medical College Hospital (Beijing, China, SYXC-2014-0029). All of the animal operations were conducted in compliance with the National Institutes of Health guide for the care and use of laboratory animals.

### Intraperitoneal Glucose Tolerance Tests (IPGTT)

The IPGTT was done in male offspring at 8, 16, 20, and 24 weeks of age. After 6-h fasting, mice were injected with a glucose load intraperitoneally (2 g/kg of body weight). Blood glucose (BG) levels were measured before, and at 30, 60, and 120 min after the injection using tail vein blood with a Contour TS glucometer (Bayer, Beijing, China). The area under the curve (AUC) of the IPGTT was calculated as previously described (12).

### Serum Biochemical Parameters Measurement

The blood samples collected from male offspring aged 24-week after 10-h fasting were centrifuged at 3,000 × g for 10 min at 4°C. ELISA kit (80-INSMSU-E01, Salem, NH, USA) was used to detect serum insulin levels. Serum total cholesterol (TC), triglyceride (TG), low-density lipoprotein cholesterol (LDL-C), high-density lipoprotein cholesterol (HDL-C), free fatty acid (FFA), alanine aminotransferase (ALT), and aspartate aminotransferase (AST) were measured using an autoanalyzer in Peking Union Medical College Hospital. Insulin sensitivity was analyzed by the homeostasis model assessment of insulin resistance (HOMA-IR). The HOMA-IR was calculated as previously described (12).

### Total RNA Extraction

We extracted total RNA from frozen liver tissues via TRIzol reagent (Life Technologies Inc., Carlsbad, CA, USA). RNA degradation and contamination were monitored on 1% agarose gels. The quality and concentration of the RNA were analyzed on a Nanodrop (ND-1000; NanoDrop Products, Wilmington, DE, USA). The RNA samples were immediately stored at −80°C for further analyses.

### Transcriptome and miRNA Sequencing

The gene expression levels of offspring liver were analyzed using whole transcriptome sequencing (*n* = 3 per group). TruSeqTM RNA sample preparation Kit from Illumina (San Diego, CA) was used for library preparation using 1 μg RNA per sample, and index codes were added. The clustering of the index-coded sequences was performed on a cBot Cluster Generation System using TruSeq PE Cluster Kit v4-cBot-HS (Illumina). After cluster generation, the library preparations were sequenced on an Illumina Hiseq X TEN and paired-end reads were generated. After quality control, the clean reads were mapped to the reference genome sequence (Mus musculus (assembly GRCm38.p6), NCBI) using Tophat2 tools soft. Differential expression analysis between two groups was performed using the DESeq R package (1.10.1). False discovery rate (FDR) <0.05 and |fold change (FC)| > 1.5 was defined as significantly differentially expressed. To discover the biological functions of the changed genes, the enrichment of genes in the KEGG (Kyoto Encyclopedia of Genes and Genomes) or Reactome pathways was analyzed using the GESA database (http://www.gsea-msigdb.org/).

To investigate the mechanism responsible for the effects of maternal exercise on intergenerational metabolic regulation at epigenetic levels, we used 3 μg total RNA per sample of offspring livers for miRNA library preparations with the NEBNext® Multiplex Small RNA Library Prep Set for Illumina® (NEB, USA.). Index codes were added to attribute sequences to each sample (*n* = 3 per group). Library quality was evaluated on the Agilent Bioanalyzer 2100 system using DNA High Sensitivity Chips. After cluster generation, the library preparations were sequenced on an Illumina Hiseq 2500 platform and single-end reads were generated. miRBase (http://www.mirbase.org/) and miRDeep2 database were used to convert raw data into recognizable miRNA expression data and annotated novel miRNA, respectively. Differentially expressed miRNAs were determined by DESeq2 R package based on the combined criteria of *p* < 0.05 and |FC| > 2. The miRanda (http://www.miranda.org), TargetScan (http://www.targetscan.org/), and PicTar (http://pictar.mdc-berlin.de) online databases were used for identifying the targets of the differentially expressed miRNAs and their overlaps were defined as the target genes of each miRNA in this study. Subsequently, their target genes were enriched in the Reactome pathway to further explore their potential biological functions.

### Reverse Transcription-Quantitative Polymerase Chain Reaction (RT-qPCR) Experiments

To validate the reliability of the whole-transcriptome sequencing results, three differentially expressed genes were chosen for RT-qPCR analysis (*n* = 6 per group). Total RNA was extracted as mentioned above. One μg of RNA was reversely transcribed into complementary deoxyribonucleid acid (cDNA) using the PrimeScript TM RT reagent Kit with gDNA Eraser (RR047A, TaKaRa Bio Inc., Otsu, Shiga, Japan). TB Green PCR Master Mix (RR820A, Takara Bio Inc., Otsu, Shiga, Japan) was used to amplify cDNA (2 μl) on an ABI 7500 thermocycler (Applied Biosystems, CA, USA) in a 20 μL reaction volume. The reaction steps included: [1] initial denaturation (30 s at 95°C); [2] cycling (denaturation for 5 s at 95°C and annealing and extending for 34 s at 60°C for 40 cycles. PPIA was used for normalization. Supplementary Table 1 listed the primer sequences. The relative expression levels of the genes were quantified by 2^−Δ*ΔCt*^ method.

### Statistics

The normality of the data was evaluated using the Kolmogorov–Smirnov test. Normally distributed continuous data were expressed as mean ± standard error (SD) and were analyzed by one-way ANOVA or two-way ANOVA, with Turkey *post hoc* analyses. Non-normally distributed continuous variables were expressed as median and interquartile range and were analyzed by the Kruskal-Wallis test. A *p*-value <0.05 was considered statistically significant. Prism version 7.0 (GraphPad Software Inc., San Diego, CA, USA) was used for statistical analysis.

## Results

### Maternal Exercise During Three-Week Prepregnancy and Throughout Pregnancy Prevented Male Offspring From Obesity Induced by Maternal HFD

As shown in Figure 1B, the body weight was dramatically higher in male offspring at the end of lactation (3rd week) in the HF group than those in the C group (*p* < 0.01), and maternal exercise significantly suppressed the increases in body weight induced by maternal HFD (*p* < 0.05). As the offspring grew to adulthood (12 and 13 weeks old), the offspring of the maternal HFD group gradually tended to become obese, whereas maternal exercise could protect offspring in the HF-EX group from obesity and even lasted to the middle-aged (24 weeks old), whose body weight was similar to that in the C group. Simultaneously, the SAT and VAT were significantly increased in offspring of the HF group than those of the C group (both *p* < 0.001). Maternal exercise dramatically decreased these two kinds of white fat mass induced by maternal HFD in adult offspring at 24 weeks (both *p* < 0.05) (Figures 1C,D).

### The Effects of Maternal HFD and Exercise on Glucose and Lipid Metabolic Parameters in Male Offspring

Since maternal HFD and exercise intervention have significant programming effects on the body weight and fat mass of the offspring, we further dynamically analyzed the effects of maternal intervention on glucose and lipid metabolism in offspring among the three groups. At 8 weeks, offspring from the HF group had impaired glucose tolerance as measured by IPGTT compared to offspring from the C group. With the growth and development of offspring to 20 weeks of age, the abnormal glucose tolerance of offspring of maternal HFD was more obvious (*p* < 0.001). In contrast, maternal exercise intervention remodeled the trajectory of adult disease caused by HFD and ameliorated glucose metabolism abnormalities in midlife (20 weeks old, *p* = 0.05), and almost normalized the blood glucose levels of offspring at 24 weeks old (Figures 1E,F). Consistent with the results of glucose tolerance, the fasting serum insulin level and HOMA-IR index of the offspring in the HF group were significantly higher than those in the C group (*p* < 0.05), revealing insulin resistance. Interestingly, maternal exercise reversed the poor “metabolic memory” caused by the harmful nutritional environment in early life and markedly improved the insulin sensitivity of adult offspring (Table 1).

**Table 1:** Serum biochemical parameters of male offspring aged 24 weeks.

**Parameters**	**C**	**HF**	**HF-EX**	**P for HF vs. C**	**P for HF-EX vs. HF**
N	6	6	6	N/A	N/A
Insulin (ng/ml)	0.12 (0.10–0.26)	0.31 (0.22–0.60)	0.13 (0.08–0.20)	0.073	0.027
HOMA-IR	0.94 (0.60–1.59)	1.68 (1.46–4.32)	0.83 (0.57–1.51)	0.061	0.035
TC (mmol/l)	2.12 ± 0.25	2.49 ± 0.19	2.15 ± 0.18	0.015	0.025
TG (mmol/l)	0.31 ± 0.07	0.45 ± 0.08	0.40 ± 0.04	0.003	0.288
LDL-C (mmol/l)	0.14 ± 0.04	0.19 ± 0.06	0.12 ± 0.03	0.123	0.038
HDL-C (mmol/l)	1.48 ± 0.14	1.20 ± 0.40	1.31 ± 0.18	0.155	0.682
FFA (nmol/l)	1276.9 ± 214.6	1311.4 ± 260.0	1350.1 ± 155.8	0.945	0.931
ALT (u/l)	38.9 ± 11.8	46.7 ± 7.3	43.2 ± 7.7	0.269	0.744
AST (u/l)	128.5 (111.9–168.1)	214.7 (187.9–246.3)	188.2 (177.2–206.8)	0.019	0.661

*Data presents as mean ± SD or median and interquartile range. One-way ANOVA and Kruskal-Wallis test were used to analyze differences among the groups. p <0.05 was considered statistically significant. C, offspring of dams fed the normal control diet; HF, offspring of dams fed the high-fat diet; HF-EX, offspring of dams intervened with a high-fat diet and exercise; HOMA-IR, homeostasis model assessment of insulin resistance; TC, total cholesterol; TG, triglyceride; LDL-C, low-density lipoprotein cholesterol; HDL-C, high-density lipoprotein cholesterol; FFA, free fatty acid; ALT, alanine aminotransferase; AST, aspartate aminotransferase; N/A, not applicable*.

In addition to glucose tolerance, the effects of maternal HFD and exercise on serum lipid profiles were also detected to evaluate the differences in lipid metabolism of male adult offspring (24-week-old). As shown in Table 1, maternal HFD throughout 3-week-prepregnancy, pregnancy, and lactation dramatically increased the serum level of TC (*p* < 0.01), TG (*p* < 0.05), and LDL-C (*p* < 0.01) in comparison with those in the C group. Prenatal 6-week exercise intervention resulted in a remarkable improvement in the serum TC and LDL-C (both *p* < 0.05). However, no significant differences in HDL-C and FFA concentrations were observed among the three groups.

### Maternal Perinatal Lifestyle Intervention Transcriptionally Regulated Gene Expressions in Adult Male Offspring at 24 Weeks

**Figure 2:**
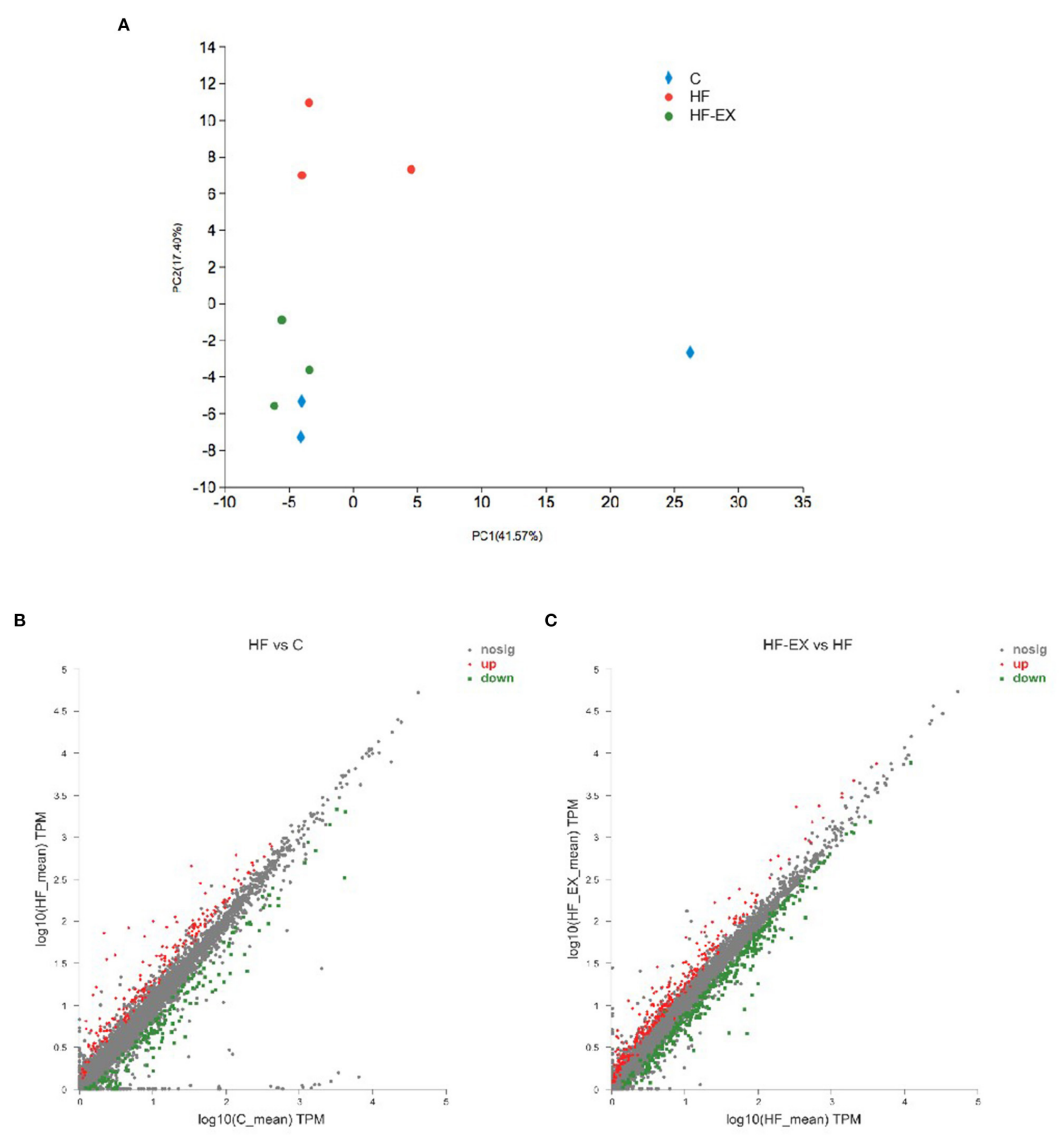
Principal component analysis and scatter plot of hepatic transcriptome sequencing in adult offspring. **(A)** Principal component analysis; **(B)** scatter plot of HF vs. C; **(C)** scatter plot of HF-EX vs. HF. C, offspring of dams fed the normal control diet; HF, offspring of dams fed the high-fat diet; HF-EX, offspring of dams intervened with a high-fat diet and exercise (*n* = 3/group).

To further reveal the effect of maternal perinatal dietary and exercise intervention on glucose and lipid metabolism at the molecular level, we performed whole transcriptome sequencing analyses for adult offspring livers, one of the most vital metabolic organs. A total of 76.47 Gb clean data were obtained from nine samples. After comparing with the reference genome, an amount of 25,480 genes were identified. Principal component analysis (PCA) demonstrated that maternal HFD and exercise intervention induced significant changes and marked separations in the hepatic gene expression of their offspring and the genes in offspring from the HF-EX group was much closer to that in the C group (Figure 2A). Figure 2B illustrated that 195 genes were up-regulated and 158 genes were down-regulated in the comparison between the HF group and the C group (FDR <0.05 and |FC| > 1.5). Meanwhile, maternal exercise led to 285 increased and 324 decreased hepatic genes compared with those of the HF group in offspring (Figure 2C).

### Functional Analysis of Altered Genes Which Were Regulated by Maternal HFD and Reversed by Maternal Exercise in Adult Offspring

**Figure 3:**
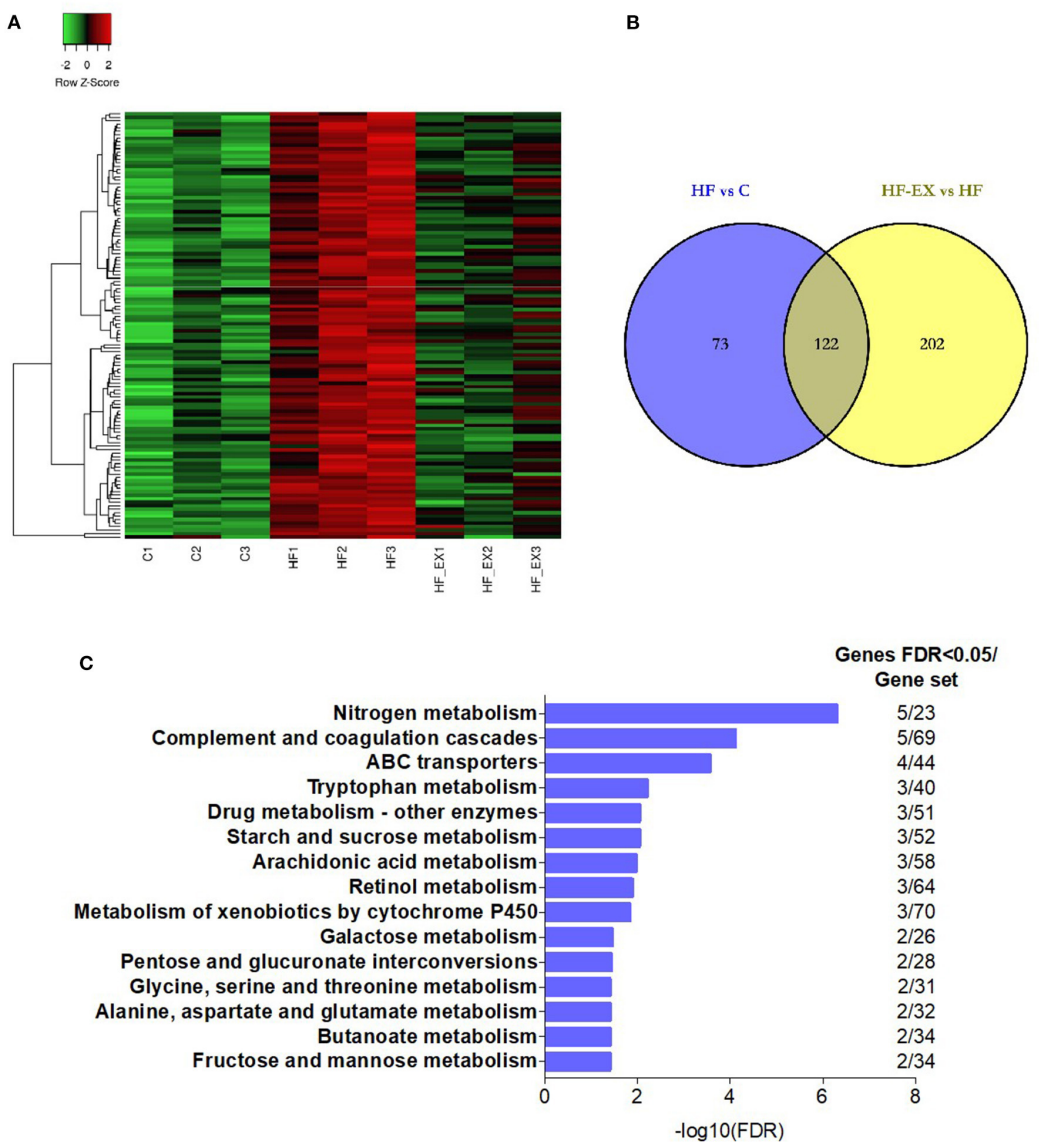
Hepatic genes that were up-regulated by maternal HFD and down-regulated by maternal exercise and their functional analysis in adult offspring. **(A)** Heatmap of the differentially expressed genes; **(B)** Venn diagram of the differentially expressed genes; **(C)** Reactome pathway enrichment of the differentially expressed genes. C, offspring of dams fed the normal control diet; HF, offspring of dams fed the high-fat diet; HF-EX, offspring of dams intervened with a high-fat diet and exercise (*n* = 3/group); HFD, high-fat diet.

To explore whether maternal exercise intervention can molecularly reverse the adverse effects of maternal HFD on offspring metabolism, we analyzed the genes that were tremendously up-regulated in the HF group but substantially down-regulated in the HF-EX group (Figure 3A). Figure 3B indicated that 122 up-regulated genes were reversed by maternal exercise intervention. These genes were significantly enriched in the signal pathways that are closely linked to glucose metabolism and amino acid metabolism such as tryptophan metabolism, starch and sucrose metabolism, arachidonic acid metabolism, retinol metabolism, galactose metabolism, and fructose and mannose metabolism (Figure 3C).

**Figure 4:**
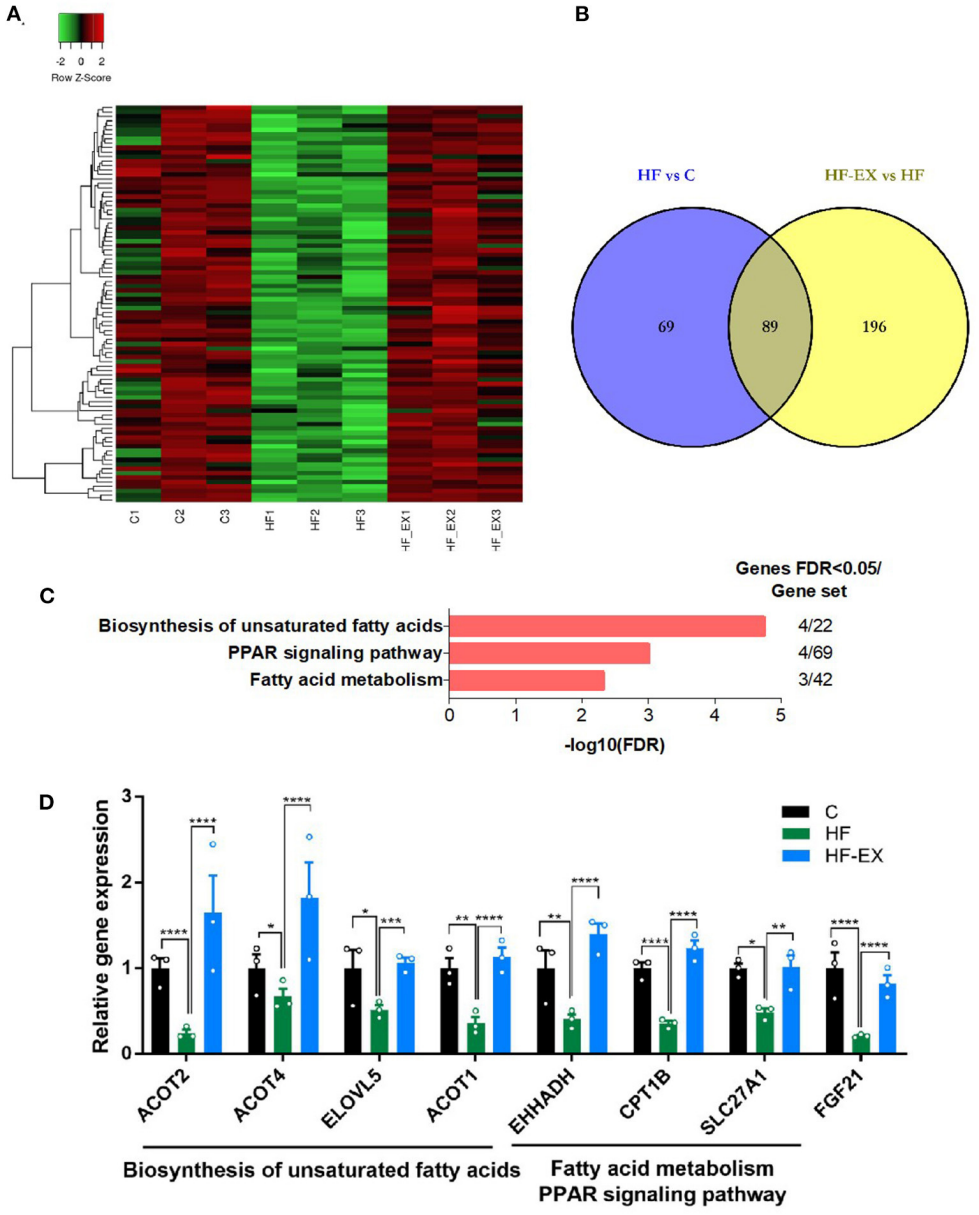
Hepatic genes that were down-regulated by maternal HFD and up-regulated by maternal exercise and their functional analysis in adult offspring. **(A)** Heatmap of the differentially expressed genes; **(B)** Venn diagram of the differentially expressed genes; **(C)** KEGG pathway enrichment of the differentially expressed genes; **(D)** differentially expressed genes. C, offspring of dams fed the normal control diet; HF, offspring of dams fed the high-fat diet; HF-EX, offspring of dams intervened with a high-fat diet and exercise; HFD, high-fat diet. Data are expressed as means ± SD (*n* = 3/group). Mean values were significantly different between the groups: *p < 0.05, **p < 0.01, ***p < 0.001, ****p < 0.0001.

On the contrary, we further analyzed genes down-regulated by maternal HFD but up-regulated by exercise intervention (Figure 4A). Eighty nine differentially expressed genes in the liver of adult offspring were positively reversed by maternal exercise intervention and were enriched in the pathways including biosynthesis of unsaturated fatty acids, PPAR signaling pathway, and fatty acid metabolism (FDR <0.05) (Figures 4B,C). Among the biosynthesis of unsaturated fatty acids pathway, *ACOT1, ACOT2, ACOT4*, and *ELOVL5* were significantly changed. Meanwhile, the expression of *EHHADH, CPT1B*, and *SLC27A1* was distinctly different among the three counterparts in the PPAR signaling pathway and fatty acid metabolism. In addition, FGF21, as a fasting-like regulator, plays a crucial role in metabolic health. Interestingly, our results demonstrated that maternal HFD significantly reduced the expression of *FGF21* in the liver of adult offspring, while maternal exercise normalized its expression level (Figure 4D). And these results were repeated by RT-qPCR and verified the reliability of our transcriptome sequencing results (Supplementary Figure 1).

### Maternal HFD and Exercise During Perinatal Period Modulated Hepatic miRNA Expression in Adult Offspring

**Figure 5:**
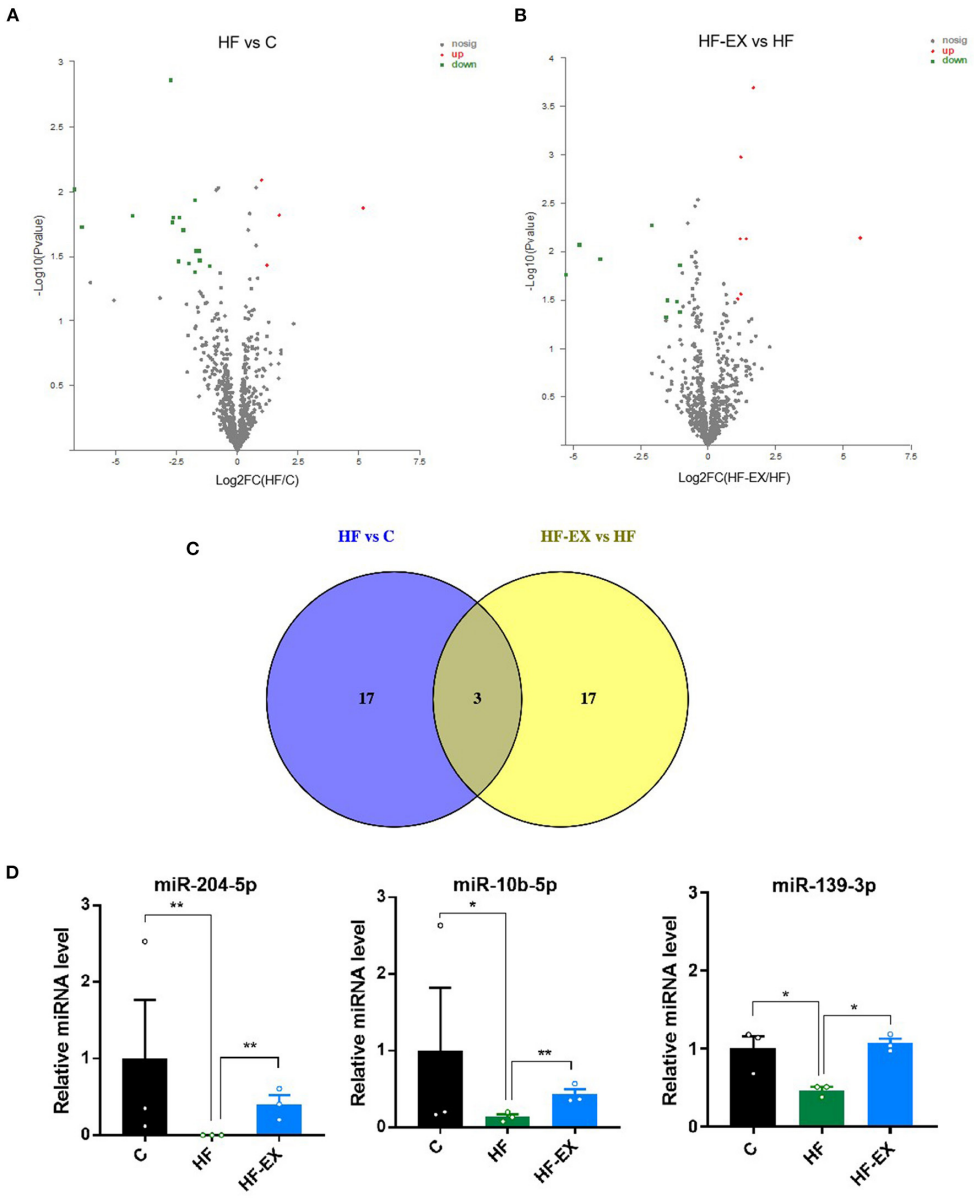
Maternal HFD and exercise regulated hepatic miRNA expression in adult offspring. **(A)** Volcano plot of miRNAs between HF and C groups; **(B)** volcano plot of miRNAs between HF-EX and HF groups; **(C)** Venn diagram of the differentially expressed miRNAs among the three groups; **(D)** lists of the three differentially expressed miRNAs. C, offspring of dams fed the normal control diet; HF, offspring of dams fed the high-fat diet; HF-EX, offspring of dams intervened with a high-fat diet and exercise; HFD, high-fat diet. Data are expressed as means ± SD (*n* = 3 / group). Mean values were significantly different between the groups: *p < 0.05, **p < 0.01.

It is apparent that maternal exercise intervention not only reversed the adverse effects of harmful intrauterine environment induced by maternal HFD on offspring metabolism, but also significantly regulated glucose and lipid metabolism at the molecular level. Then, we performed miRNA sequencing in adult offspring livers at 24 weeks to detect whether epigenetics plays an important role in the process. Twenty altered miRNAs, including 4 significantly up-regulated and 16 down-regulated miRNAs, between HF and C group were shown in Figure 5A. On the other hand, maternal exercise sharply raised 7 and declined 13 miRNA expression levels compared with those in the HF group (*p* < 0.05 and |FC| > 2, Figure 5B). Figure 5C visualized that there were three common differentially expressed miRNAs between HF vs. C and HF-EX vs. HF, including miR-204-5p, miR-10b-5p, and miR-139-3p. They were all significantly down-regulated by maternal HFD and markedly up-regulated by maternal exercise in livers of offspring (Figure 5D).

### Functional Enrichment Analysis for the Target Genes of the Differentially Expressed miRNAs

**Figure 6:**
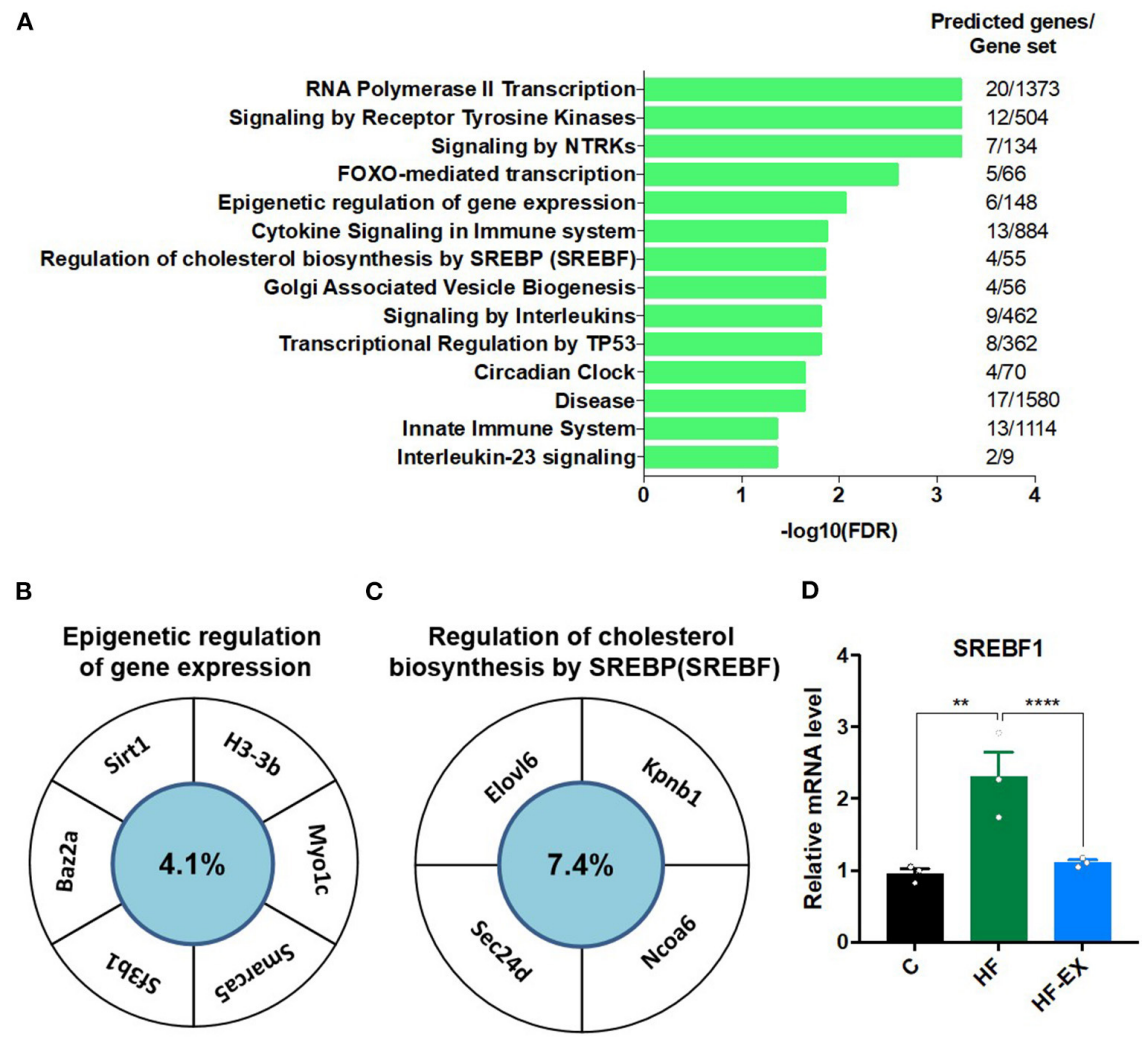
Analysis of the targeted genes of the differentially expressed hepatic miRNAs in adult offspring regulated by maternal exercise. **(A)** Reactome pathway enrichment analysis of the target genes; **(B)** genes enriched in the epigenetic regulation of gene expression pathway; and **(C)** target genes involved in the regulation of cholesterol biosynthesis by SREBP pathway; **(D)** *SREBF1* expression level. C, offspring of dams fed the normal control diet; HF, offspring of dams fed the high-fat diet; HF-EX, offspring of dams intervened with a high-fat diet and exercise; SREBF1, sterol regulatory element binding protein-1. Data are expressed as means ± SD (*n* = 3/group). Mean values were significantly different between the groups: **p < 0.01, ****p < 0.0001.

Finally, we analyzed the target gene functions of the three miRNAs. The predicted genes of miR-204-5p, miR-10b-5p, and miR-139-3p were overlapped by TargetScan, miRanda, and PicTar (Supplementary Table 2). Figure 6A showed that their target genes were enriched in the signaling by receptor tyrosine kinases, FOXO-mediated transcription, epigenetic regulation of gene expression, cytokine signaling in immune system, regulation of cholesterol biosynthesis by SREBF, circadian clock, interleukin-23 signaling, and some other pathways (FDR <0.05). Given the vital role of epigenetic regulation of gene expression pathway and regulation of cholesterol biosynthesis by SREBF pathway in metabolism, we focused on genes that may be regulated by miRNAs in these two signaling pathways. *SIRT1, H3-3B, MYO1C, SMARCA5*, and *BAZ2A* were enriched in the signaling pathway of epigenetic regulation (Figure 6B), and *ELOVL6, KPNB1, NCOA6*, and *SEC24D* were classified into cholesterol synthesis signal pathway (Figure 6C). We further checked the expression level of *SREBF1* from the results of hepatic transcriptome sequencing and found that maternal HFD significantly increased *SREBF1* expression in adult offspring, while maternal exercise dramatically decreased its level (Figure 6D).

## Discussion

Maternal adverse intrauterine environment is a well-recognized factor for the development of metabolic disturbances in offspring as they age, initiating a vicious circle that likely contributes to the sharp surge in rates of obesity and T2DM (2–9). Consistently, our results also confirmed that maternal HFD throughout 3-week-prepregnancy, gestation, and lactation programmed glucose intolerance, insulin resistance, and dyslipidemia in adult male offspring. Exercise intervention is often recommended as the cornerstone of diabetes care, but its intergenerational metabolic protective effect is poorly understood, in part due to the difficulty in performing long-term follow-up in human cohort studies (24). Fortunately, several literatures have reported that maternal exercise results in metabolic benefits to offspring from young into adulthood by taking the advantage of animal models (11, 12). In the present investigation, we carried out a VWR training 3-week before breeding and during pregnancy in HFD-fed mothers and found that maternal exercise protected offspring from obesity. At the same time, it significantly improved the glucose tolerance and insulin sensitivity of male adult offspring, reduced their body fat content, and ameliorated dyslipidemia. These data demonstrated that exercise intervention in the early stage of life can effectively improve and even reverse the adverse “metabolic memory” induced by maternal HFD in adulthood, and timely reduce or block the occurrence of metabolic disorders in the future.

There might be numerous underlying mechanisms responsible for the effects of maternal exercise on reprogramming the health and disease trajectory of offspring. Several laboratories have observed that maternal exercise (treadmill or VWR) before conception and during pregnancy increased glucose uptake of skeletal muscles and adipose tissues, decreased glucose production hepatocytes, accelerated energy expenditure of brown adipose tissue, and simultaneously reduced the size of pancreatic β-cells and insulin secretion in offspring (10). As the liver is a major regulator of whole-body metabolic homeostasis, we hypothesized that gene expressions in the liver of offspring were altered due to different maternal lifestyles. Indeed, the results of whole transcriptome sequences in adult male offspring showed that 9-week maternal HFD feeding resulted in great changes in the gene expression level compared with that in the control group, whereas exercise training of dams 3 weeks before mating and during pregnancy reversed these detrimental effects, especially the genes involved in the pathways of glucose and amino acid metabolism, biosynthesis of unsaturated fatty acids, fatty acid metabolism, and PPAR signaling pathway.

Environmental factors and physical activity have been proved to regulate epigenetic modification, which could be a crucial reason for deciphering their metabolic benefits (25–27). Given the critical role of epigenetics in the link of adverse early-life environments with adult metabolic disorders, we analyzed the expression level of miRNAs in the liver of adult offspring and found that miR-204-5p, miR-10b-5p, and miR-139-3p were significantly down-regulated in the maternal HFD group, while maternal exercise up-regulated their expression. After target gene prediction and signal pathway enrichment analysis, a striking finding was that the differentially expressed miRNAs participated in the lipid metabolism and epigenetic regulation, which might explain the pronounced effect of maternal exercise intervention on adult offspring metabolism.

miR-204-5p is a tumor suppressor and has been widely confirmed to inhibit the growth and metastasis of melanoma, breast cancer, osteosarcoma, and other tumors (28–31). In terms of metabolism, Grieco et al. (32) found that pro-inflammatory cytokines interleukin 1 β (IL1β) and interferon γ (IFNγ) could trigger endoplasmic reticulum (ER) stress and the expression of pro-apoptotic members of the BCL2 protein family in β-cells, thus contributing to their death. Further miRNA expression profile analysis observed a cytokine-induced down-regulation of miR-204-5p, and inhibitor blocking endogenous miR-204-5p increased β-cell apoptosis and induced ER stress. These observations identify novel crosstalk between miR-204-5p, ER stress, and β-cell inflammation and apoptosis. Moreover, prior studies have noted the importance of miR-204-5p in the protection of renal function. Cheng et al. (33) reported that kidneys of patients with hypertensive nephrosclerosis or diabetic nephropathy exhibited a marked decrease in miR-204-5p compared with healthy subjects. In diabetic db/db mice and the mouse model of hypertensive renal injury induced by uninephrectomy, angiotensin II, and a high-salt diet, inhibiting miR-204-5p or deleting miR-204 led to exacerbated albuminuria, renal interstitial fibrosis, and interlobular artery thickening. Another study also mentioned that increased miR-204-5p might suppress renal ischemia-reperfusion injury in mice by suppressing Fas/FasL pathway (34). In addition, a strong relationship between miR-204-5p and adipose tissue and skeletal muscle metabolism has been reported in the following literatures: promoting lipid synthesis by targeting *SIRT1* (35), adjusting 3T3-L1 preadipocyte proliferation by negatively regulating *KLF3* (36), controlling adipogenesis in human mesenchymal stem cells by regulating *DVL3* expression and subsequently inhibiting the activation of the Wnt/β-catenin signaling pathway (37), and changing skeletal muscle mitochondrial oxidative capacity by stimulating its biogenesis (38). As for the liver, only one study indicated that the overexpression of miR-204-5p in human hepatocellular carcinoma cell lines targeted *SIRT1* and caused the suppression of cell survival and the increase of apoptosis and drug sensitivity (39). Hence, our study confirmed for the first time in the animal model that maternal exercise can reverse the lowered expression level of miR-204-5p in the liver of offspring induced by maternal HFD, and may play an important role in mediating the intergenerational improvement of metabolism.

miR-10b-5p is a novel miRNA that has attracted growing attention in recent years. Emerging evidence suggested that it acted as a biomarker of diagnostic and prognostic for breast cancer, gastric cancer, and other malignant tumors (40, 41). When it comes to metabolism, studies revealed that miR-10b-5p antagonized hypoxia-induced cardiomyocyte apoptosis (42), fine-tuned 3T3-L1 pre-adipocytes differentiation by targeting *APOL6* (43), and got involved in fatty acid metabolism (44). Iacomino et al. subsequently demonstrated a remarkable reduced level of circulating miR-10b-5p in obese children, which further confirmed its important role in the regulation of metabolism (45). Besides, miR-10b-5p is also a novel T helper cell 17 (Th17) regulator (46). However, very few literatures focused on the role of miR-10b-5p in the liver, with the exception of one study identifying a significant change of miR-10b-5p levels in patients with hepatitis B virus infection-induced liver fibrosis through liver biopsy and miRNAs detection (47). So far, the data exploring the role of miR-10b-5p in mediating “metabolic memory” is scarce. To the best of our knowledge, this is the first study to find that exercise intervention started from three weeks prior to mating and lasted throughout pregnancy markedly elevated the expression level of miR-10b-5p in adult offspring liver, which was accompanied by the improvement of lipid and glucose metabolism. These outcomes extended the understanding of miR-10b-5p in mediating the programming effects of maternal exercise intervention on adult offspring hepatic metabolism.

Similarly, miR-139-3p has gained wide attention in the field of oncology. There has been increased recognition that miR-139-3p could suppress the invasion and migration properties of breast cancer cells by targeting *RAB1A* (48), inhibit metastasis of cervical cancer by targeting *NOB1* (49), restrain the proliferation, invasion, and migration of human glioma cells by targeting *MDA-9*/*syntenin* (50), and be used as a prognostic marker for a variety of tumors (51). In regards to metabolism, Wang et al. found that miR-139-3p could target *ELK1*, and then regulate osteoblast differentiation and apoptosis (52); using a diabetic rat model after sleeve gastrectomy, findings from Li et al. interpreted a possible relationship between hepatocyte apoptosis and miR-139-3p expression level (53). Additionally, miR-139 also contributes to the protection against drug-induced myocardial ischemia-reperfusion injury (54). Unfortunately, the regulation role of miR-139-3p in hepatic metabolism has not been revealed. Our work filled this gap, provided an important contribution to the field of miR-139-3p in DOHaD theory, and indicated its expression level elevated in the adult offspring liver from exercise-trained mothers.

In conclusion, we demonstrated that maternal exercise programmed glucose and lipid metabolism at both phenotypic and molecular levels in adult offspring. Moreover, for the first time to our knowledge, this study clarified the role of miRNAs in mediating the programming effects of maternal exercise on adult offspring metabolism, and found that maternal exercise before and during pregnancy resulted in a significant increase in miR-204-5p, miR-10b-5p, and miR-139-3p in offspring livers, which were decreased by maternal HDF feeding. And these miRNAs took part in cholesterol synthesis regulated by SREBF and epigenetic regulation. Taken together, this study implicated that miRNA might be another critical contributor to the intergenerational metabolic benefits of maternal exercise, which might indicate novel targets for fighting against metabolic disorders and provide insight into the prevention and treatment of diabetes in the early life stage. However, future research is also required to determine the specific mechanism of the differentially expressed miRNAs in mediating the intergenerational metabolic regulation of maternal exercise, and address questions of the role of epigenetics in “metabolic memory.”

## Data Availability Statement

The datasets presented in this study can be found in online repositories. The names of the repository/repositories and accession number(s) can be found below: https://www.ncbi.nlm.nih.gov/, PRJNA796070.

## Ethics Statement

The animal study was reviewed and approved by the Animal Care and Use Committee of the Peking Union Medical College Hospital (Beijing, China, SYXC-2014-0029).

## Author Contributions

LZ was responsible for the animal experiments, data analysis, making figures, tables, drafting, and revising the manuscript. SL drafted part of the manuscript. QZ and MY helped with the data analysis and study design. XX contributed to the whole study design, data interpretation, and reviewed the manuscript. All authors approved the final version of this manuscript.

## Funding

This work was supported by the grants from National Natural Science Foundation of China (Nos. 82170854, 81870579, 81870545, 81570715, and 81170736), Beijing Natural Science Foundation (No. 7202163), Beijing Municipal Science & Technology Commission (No. Z201100005520011), National Key Research and Development Program of China (No. 2018YFC2001100), and CAMS Innovation Fund for Medical Sciences (Nos. CIFMS2021-1-I2M-002 and CIFMS2017-I2M-1-008).

## Conflict of Interest

The authors declare that the research was conducted in the absence of any commercial or financial relationships that could be construed as a potential conflict of interest.

## Publisher's Note

All claims expressed in this article are solely those of the authors and do not necessarily represent those of their affiliated organizations, or those of the publisher, the editors and the reviewers. Any product that may be evaluated in this article, or claim that may be made by its manufacturer, is not guaranteed or endorsed by the publisher.
