# Supplementary material for: Maternal Exercise Programs Glucose and Lipid Metabolism and Modulates Hepatic miRNAs in Adult Male Offspring
Source: Front Nutr. 2022 Mar 1;9:853197. doi: 10.3389/fnut.2022.853197 (PMC8923645; doi:10.3389/fnut.2022.853197)
Supplement: Supplementary file 1 [file Table_1.DOCX]

Supplementary Material

# Supplementary Figures and Tables

## Supplementary Figures

**Figure S1.** RT-qPCR validation of the selected genes which were significantly different among the three groups in the transcriptome results. C, offspring of dams fed the normal control diet; HF, offspring of dams fed the high-fat diet; HF-EX, offspring of dams intervened with a high-fat diet and exercise. Data are expressed as means ± SD (n = 6 / group) and are analyzed by one-way ANOVA. Mean values were significantly different between the groups: *p<0.05, **p<0.01, ***p<0.001.


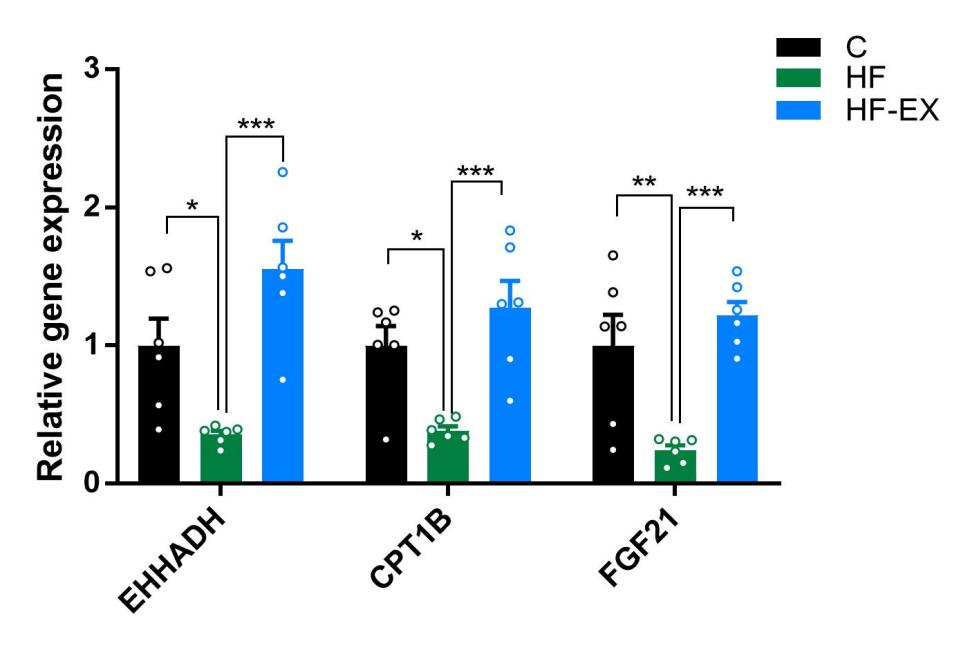


**1.2 Supplementary Tables**

**Table S1.** Primer sequences for *EHHADH, CPT1B, FGF21*, and *PPIA.*

| **Genes** | **Primer sequence** |
| --- | --- |
| ***EHHADH*** | F-ATGGCTGAGTATCTGAGGCTG; R-GGTCCAAACTAGCTTTCTGGAG |
| ***CPT1B*** | F-GCACACCAGGCAGTAGCTTT; R-CAGGAGTTGATTCCAGACAGGTA |
| ***FGF21*** | F-GTGTCAAAGCCTCTAGGTTTCTT; R-GGTACACATTGTAACCGTCCTC |
| ***PPIA*** | F-GCTGGACCAAACACAAACGG; R-TCCTGGACCCAAAACGCTC |

**Table S2.** The predicted genes of miR-204-5p, miR-10b-5p and miR-139-3p overlapped by TargetScan, miRanda and PicTar.

| **miRNAs** | **Predicted genes** |
| --- | --- |
| **miR-204-5p** | *FAM168B, SF3B1, SATB2, AP1S3, PID1, TRIP12, BCL2, CCNT2, SIRT1, GUCD1, FRS2, HMGA2, IL23A, LATS1, ATP2B1, BAZ2A, CPD, AKAP1, P4HB, RAB1, MYO1C, RHOT1, SPOP, SOCS7, TANC2, RAB10, SOX11, ZFP36L1, RPS6KA5, RIT1, SMOC1, EEF1E1, RHOBTB3, LARP4B, FOXC1, RREB1, NTRK2, ELL2, AP3M1, DLG5, FARP1, ANGPT1, HAS2, RICTOR, MYO10, 1810013L24RIK, DVL3, EZR, IGF2R, BIRC6, KCTD1, NR3C1, MAPRE2, MAPRE2, ZFP91, KMT5B, GNAQ, SEC61A2, ATF2, NDRG3, SERINC3, NRARP, COL5A1, FUBP3, SSRP1, SPRED1, CHP1, ELF2, NBEA, PRPF38B, HS2ST1, ZFHX4, MBNL1, ARHGAP29, SEC24D, ELOVL6, LPAR1, PTPRD, KHDRBS1, SZRD1, TRP53INP1, AK4, SSBP3, RERE, CHD5, ARAP2, DTX1, AP1S1, KLF3, FRAS1, PCGF3, ANKRD13A, TRIAP1, SLC37A3, HNRNPA2B1, PPM1K, CHN2, ITPR1, ARL8B, FAM160A2, TTYH1, GABRB3, WEE1, ABRAXAS2, SMARCA5, IRF2BP2, GPM6A, NFATC3, ZFHX3, ELAVL3, TCF12, TMOD3, SYNCRIP, PIK3CB, DNAJC13, TGFBR2, COX5A, CCPG1, GM44503, RAP2C, SLITRK4, ARX* |
| **miR-10b-5p** | *GPM6B, MYBL1, SMAP1, EPHA4, TMEM183A, RGS8, ESRRG, SYNE1, DAZAP1, MTMR3, KPNB1, H3F3B, FXR2, ANKFY1, SDC1, ZFP367, BICD2, ARRDC3, SLC38A2, BCL6, SON, ZFP608, RBM27, CELF2, NCOA6, MAPKBP1, BCL2L11, STK4, KLF4, ERI3, CTNNBIP1, EPHA5, HOXA1, LHFPL4, NACC1, PAPD5, PRTG, DOCK11* |
| **miR-139-3p** | *EIF4B* |
